# Cost-Effective Production of ATP and S-Adenosylmethionine Using Engineered Multidomain Scaffold Proteins

**DOI:** 10.3390/biom11111706

**Published:** 2021-11-17

**Authors:** Guangbo Yan, Xia Li, Jun Yang, Zhongchen Li, Jia Hou, Ben Rao, Yong Hu, Lixin Ma, Yaping Wang

**Affiliations:** 1 State Key Laboratory of Biocatalysis and Enzyme, Engineering Hubei Collaborative Innovation Center for Green Transformation of Bio-Resources, Hubei Key Laboratory of Industrial Biotechnology, Biology Faculty of Hubei University, Hubei University, Wuhan 430062, China; 17607164502@163.com (G.Y.); 15225397426@163.com (X.L.); youcojun@outlook.com (J.Y.); lizc0408@163.com (Z.L.); hj19980608@163.com (J.H.); malixing@hubu.edu.cn (L.M.); 2 National Biopesticide Engineering Technology Research Center, Hubei Biopesticide Engineering Research Center, Hubei Academy of Agricultural Sciences, Biopesticide Branch of Hubei Innovation Centre of Agricultural Science and Technology, Wuhan 430064, China; raoben1983729@aliyun.com; 3 Key Laboratory of Fermentation Engineering (Ministry of Education), Hubei Key Laboratoy of Industrial Microbiology, National “111” Center for Cellular Regulation and Molecular Pharmaceutics, Hubei Research Center of Food Fermentation Engineering and Technology, Hubei University of Technology, Wuhan 430062, China; huyonghugong@163.com

**Keywords:** S-adenosylmethionine, ATP synthesis, scaffold protein, multi-enzyme cascade

## Abstract

Adenosine triphosphate (ATP) and S-adenosyl-L-methionine (SAM) are important intermediates that are widely present in living organisms. Large-scale preparation and application of ATP or SAM is limited by expensive raw materials. To lower the production costs for ATP/SAM, in this study we used strategies applying engineered multidomain scaffold proteins to synthesize ATP and SAM. An artificial scaffold protein containing CBM3 domain, IM proteins and CL-labeled proteins was assembled to form complex 1 for catalytic reactions to increase ATP production. The ATP synthesis system produced approximately 25 g/L of ATP with approximately 15 g/L of ADP and 5 g/L of AMP using 12.5 g/L of adenosine and 40 g/L of sodium hexametaphosphate reaction at 35 °C and a pH of 8.5 for 6 h. Based on the above ATP synthesis system, two CL-labeled methionine adenosyltransferases (CL9-MAT4 and CL9-MAT5) were applied to construct scaffold protein complex 2 to achieve SAM synthesis. Approximately 25 μg of MAT4 in a reaction system with 0.3 M MgCl_2_ catalyzed at 20 °C and a pH of 8 catalyzed 0.5 g/L of l-Met to produce approximately 0.9 g/L of SAM. Approximately 25 μg of MAT5 in a reaction system with 0.7 M MgCl_2_ catalyzed at 35 °C and a pH of 8 catalyzed 0.5 g/L of l-Met to produce approximately 1.2 g/L of SAM. Here, we showed that low-cost substrates can be efficiently converted into high-value additional ATP and SAM via multi-enzyme catalytic reactions by engineered multidomain scaffold proteins.

## 1. Introduction

Adenosine triphosphate (ATP), an important metabolite in living organisms, serves as a metabolic intermediate, coenzyme, and energy donor. ATP also participates in a variety of biochemical reactions, thus providing a direct source of energy required for life. As a coenzyme drug, ATP can treat several diseases, such as heart-related diseases, filamentous keratitis, stroke sequelae, gastroptosis, and tumors [1]. ATP also has considerable application value in environmental protection [2] and microbiological testing. Researchers have made continuous efforts to synthesize ATP in vitro. Specifically, some researchers have used adenylate kinase and acetate kinase coupling to convert adenosine to ATP [3]. Kim et al. [4] also used acetyl phosphate to convert adenosine into ATP under the combined action of three enzymes (adenosine kinase, adenylate kinase, and acetate kinase). Akihiko et al. [5] used adenine as a substrate to synthesize ATP and reported that the ATP product amount reached 117 mmol/L and the substrate conversion rate reached 82%.

ATP is involved in the synthesis of many important substances. The enzymatic method uses ATP as a raw material to produce pharmaceutical chemicals and has reached the industrial application scale, such as the production of S-adenosyl-L-methionine (SAM). SAM is an important metabolic intermediate widely present in biological cells that acts as an activation group donor in a series of metabolic reactions [6,7]. It is also precursor or synthetic substrate of important substances, such as cysteine, taurine, glutathione, and coenzyme A [6,8]. SAM can be used to treat depression [9,10], osteoarthritis, tumors [11,12], and hepatobiliary diseases [13]. Rountree et al. [14] knocked out the mouse MAT gene and found that cancer cell genes were highly expressed, thus confirming that SAM has an inhibitory effect on cancer cells. It has been reported that SAM can be produced by changing the l-Met supply level to optimize fermentation conditions. In recent years, there have been some reports on SAM production [15]. Gross et al. [16] isolated SAM synthetase from *Saccharomyces cerevisiae* and successfully used SAM synthetase in a catalytic reaction. Microorganisms have been applied to produce SAM in previous reports. Markham et al. [17] constructed recombinant *Escherichia coli* containing the metK gene and increased the expression of SAM synthetase 80-fold. Yu et al. [18] constructed a recombinant strain expressing metK in *E. coli*; 1 g/L of l-Met produced 128.2 mg/L of SAM in a shake flask. In addition, the highest SAM yield was 300.9 mg/L after 8 h of induction with a 5-L starter. Hui et al. [19] optimized the l-Met feeding strategy, finding that continuous feeding facilitated SAM accumulation, and the output reached 8.46 g/L.

Recently multienzymatic systems have become important tools for synthesis of many chemicals. For example, a modular one-pot four-enzyme cascade with a PEP-based phosphate donor recycling system was constructed and used to efficient produce a wide range of NTPs [20]. In another study, researchers developed a novel one-pot enzyme cascade containing adenine phosphoribosyltransferase (APT), polyphosphate kinase (PPK), and ribonucleotide reductase (RNR), which was applied to biosynthesize cladribine triphosphate successfully [21]. In addition, a review report demonstrated that in vitro multienzymatic systems have been widely used for the synthesis of NADs production which can replace traditional chemical methods [22]. Moreover, some of the multienzymatic systems were designed as multidomain scaffold proteins to optimize enzyme reaction rate and reaction efficiency. Dueber et al. constructed a synthetic scaffold consisting of three domains (GBD, SH3 and PDZ) to produce mevalonate and achieved a 1.4-fold increase. Using similar multidomain scaffolds, researchers synthesized glucaric acid, resveratrol, butyrate, and L-serine. This technology showed great potential in the biosynthesis industry [23].

In this study, we tried to produce ATP and SAM by engineered multidomain scaffold proteins. First, an artificial scaffold protein containing the CBM3 domain, IM protein region (IM2, IM7 [24], IM8, IM9) and CL-labeled proteins (CL7-ADK; CL2-AK (mut); and CL8-PAP) were assembled to form complex 1 for catalytic reactions to increase ATP production. This scaffold protein produced approximately 25 g/L of ATP with approximately 15 g/L of ADP and 5 g/L of AMP using 12.5 g/L of adenosine and 40 g/L of sodium hexametaphosphate reaction at 35 °C and a pH of 8.5 for 6 h. Based on the above ATP synthesis system, two CL-labeled methionine adenosyltransferases (CL9-MAT4 and CL9-MAT5) were applied to construct scaffold protein complex 2 to achieve SAM synthesis. Approximately 25 μg of MAT4 in a reaction system with 0.3 M MgCl_2_ at 20 °C and a pH of 8 catalyzed 0.5 g/L of l-Met to produce approximately 0.9 g/L of SAM. Approximately 25 μg of MAT5 with 0.7 M MgCl_2_ at 35 °C and a pH of 8 catalyzed 0.5 g/L of l-Met to produce approximately 1.2 g/L of SAM. Here, we showed that low-cost substrates can be efficiently converted into high-value additional ATP and SAM by engineered multidomain scaffold proteins.

## 2. Materials and Methods

### 2.1. Genes, Strains and Reagents

The expression strain, *E. coli* BL21PlysS, was purchased from Invitrogen (Shanghai, China). The expression strain, *E. coli* BL21 (DE3), the cloned strain, *E. coli* DH5α, and plasmids (pET-23a(+), pET-28a(+), and pET23a-IM2-IM7-IM8-IM9) were all maintained in our laboratory. 5’-adenosine triphosphate, 5’-adenosine diphosphate, adenine nucleoside, sodium hexametaphosphate, and l-Met were purchased from Shanghai Maklin Biochemical Technology Co., Ltd. (Shanghai, China). *Bam*H I and *Eco*R I were purchased from NEB (Shanghai, China).

### 2.2. Recombinant Plasmid Construction

Adenosine kinase ([AK], NP_001040165.1), adenylate kinase ([ADK], AAC44863.1), phosphate: AMP phosphotransferase gene ([PAP], AGV62123.1), methionine adenosyltransferase (MAT), MAT4 (NP_417417.1), and MAT5 (WP_038038694.1) were all synthesized by Wuhan GeneCreate Biological Engineering Co., Ltd. (Wuhan, Hubei, China). We will describe the IM and CL proteins in detail in a future article. As the CL tag protein can promote solubility and expression, and may improve recombinant protein heat stability [24], the target gene fragment was amplified by overlapping PCR and connected to the CL tag protein gene fragment. Subsequently, the T5 exonuclease-mediated cloning method was used to assemble ADK, PAP, and MAT-related gene fragments with pET-23a(+) vector cut by *Bam*H I and *Eco*R I. The AK-related gene fragment was ligated to pET-28a(+) and cut by *Bam*H I and *Eco*R I to construct recombinant plasmids (pET28a-CL2-AK, pET23a-CL7-ADK, pET23a-CL8-PAP, pET23a-MAT4, pET23a-MAT5, pET23a-CL9-MAT4 and pET23a-CL9-MAT5) with a 6× His tag at the C-terminus. Song et al. [25] suggested that a single-base mutation at the S210A site could increase the activity of AK from this species. Therefore, using plasmid pET28a-CL2-AK as a template, site-directed mutagenesis was used to construct S210A site mutation mutant. The recombinant plasmid was designated pET28a-CL2-AK (mut). The primers used in the experiments were synthesized by Shanghai Sangon Biological Engineering Technology & Services Co., Ltd. (Shanghai, China).

### 2.3. Protein Expression and Purification

The plasmids (pET23a-CL7-ADK, pET23a-CL8-PAP, pET23a-MAT4, pET23a-MAT5, pET23a-CL9-MAT4, pET23a-CL9-MAT5, and pET23a-IM2-IM7-IM8-IM9) were transformed into *E. coli* BL21(DE3), which were cultivated in 100 μg/mL of ampicillin LB medium (5 g/L of yeast extract, 10 g/L of tryptone, and 10 g/L of NaCl) to an OD_600_ of 0.6–0.8, then 1 mmol/L of isopropyl-β-D-thiogalactoside (IPTG) was added for approximately 12 h to induce expression at 220 rpm and 18°C. Similarly, the pET28a-CL2-AK (mut) plasmid was transformed into *E. coli* BL21PlysS. After induction, the cells were collected by centrifugation at 7500× *g* for 5 min, then resuspended 1–2 times in 30 mL of lysis buffer (100 mM NaCl and 100 mM Tris-HCl (pH 8.5)) for use.

The cells were resuspended and 1% protease inhibitor PMSF (Phenylmethylsulfonyl fluoride) was added according to the volume ratio. The cells were lysed with a high-pressure homogenate crusher or sonicator (400 W for 15 min) and centrifuged at 9000× *g* for 20 min. The supernatant was collected and filtered through a packed, pretreated Ni-NTA purification column, then placed on a silent mixer to allow the target protein to bind with Ni beads for approximately 1 h. Lysis buffer containing low-concentration imidazole (5–10 times volume) was used to elute the contaminated protein and an elution buffer (2–3 times the column volume) containing high-concentration imidazole to elute the enzyme. The eluted fraction was separated by 12% SDS-PAGE. The storage buffer (200 mM NaCl and 100 mM Tris-HCl (pH 8.5)) was ultra-filtrated to remove the imidazole and concentrate the protein. Finally, the protein concentration was determined using the Bradford method. The concentrated enzyme solution after the liquid exchange was divided into aliquots, quick-frozen with liquid nitrogen, and stored at −80 °C.

### 2.4. The Synthesis of ATP and SAM by Multidomain Scaffold Proteins (Un-Published in Our Lab)

**Figure 1:**
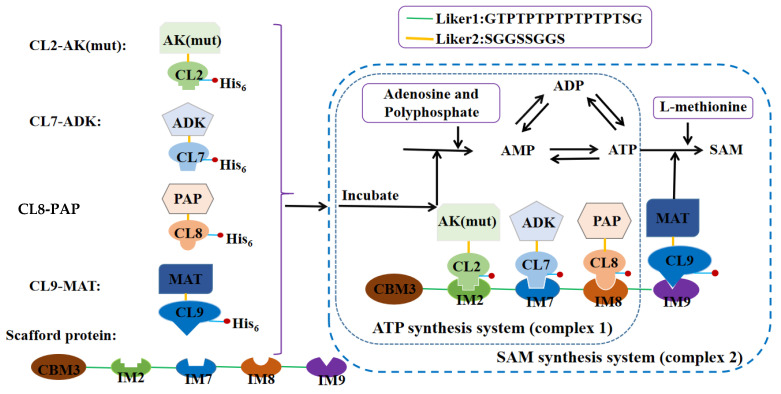
ATP and SAM synthesis system.

The ATP synthesis scaffold protein was built as follows (Figure 1): the CBM3 domain gives the scaffold protein the ability to bind to cellulose. The IM protein is conjugated with CBM3 domain via linkers. All the enzymes were fused with a CL tag. Using its specific binding with the scaffold protein, the enzymes were assembled with the scaffold protein to form a complex. Following assembly, multi-enzyme complex 1 for synthesizing ATP and multi-enzyme complex 2 for synthesizing SAM were obtained.

**Figure 2:**
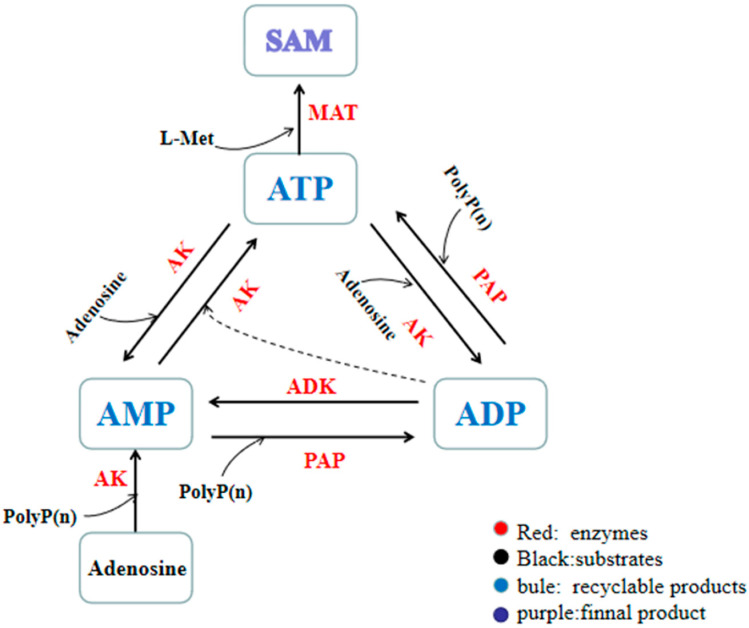
The SAM synthesis module is built on the basis of the ATP synthesis module.

Using adenosine and sodium hexametaphosphate as substrates, ATP was synthesized by the ATP synthesis module (Figure 1). Figure 2 illustrates how this ATP regeneration system works.

The ATP synthesis reaction system was as follows: 40 g/L of sodium hexametaphosphate; 12.5 g/L of adenosine; 0.1 M MgCl_2_; 0.15 M KCl; 0.35 M NH_4_Ac; 0.15 M Tris-HCl (pH 8.5); CL7-ADK; and CL8-PAP. The total volume of the reaction mixture was 300 μL, and the reaction was carried out at 35 °C. After the reaction was completed, 1:30 (*v*:*v*) 6 M HCl was added to terminate the reaction. The ATP synthesis module synthesizes ATP using adenosine and polyphosphate as the substrate, and this part of the product can be recycled.

Based on the ATP synthesis system, l-Met and CL-labeled MAT were introduced to produce SAM (Figure 1). The reaction system (the product mixture was catalyzed in the ATP synthesis system) was as follows: l-Met; 0.7 mol/L of MgCl_2_ was added to catalyze the reaction at 35 °C for 3 h (MAT5); or 0.3 mol/L of MgCl_2_ was added to catalyze the reaction with MAT4 at 20 °C for approximately 9 h. The total volume of the reaction mixture was 300 μL. After the reaction was complete, 1:30 (*v:v*) 6 mol/L of HCl was added to terminate the reaction.

### 2.5. Assembly of Enzyme and Scaffold Protein

The CBM3 domain and the enzyme components CL7-ADK, CL2-AK (mut) and CL8-PAP are mixed in equimolar amounts and maintained at room temperature for about 20–30 min to obtain ATP production multienzyme complex 1. Correspondingly, the five components of complex 1 and CL9-MAT were mixed and incubated at room temperature for 20–30 min to obtain multienzyme complex 2, which can be used to produce SAM. As the artificial scaffold protein is fused with CBM3, it gives the artificial scaffold protein [26,27] the ability to specifically bind to cellulose. In this experiment, the mixture sample was mixed with cellulose beads and combined on a silent mixer at 4 °C for about 1 h. 

In order to test the artificial scaffold protein, the protein–cellulose mixture was harvested and centrifuged at 10,000× *g* for about 1 min. The supernatant was removed and PBS buffer was added and mixed. The process was repeated for 3–5 times to remove the free enzyme components. Only when the free enzyme component is combined with the artificial scaffold protein can the corresponding protein band be detected in SDS-PAGE.

### 2.6. Detection and Analysis by High-Performance Liquid Chromatography (HPLC)

All samples were diluted 6–10 times, centrifuged at 10,000× *g* for 10 min, and the supernatant was filtered through a 0.22 μm filter into a liquid chromatography bottle for analysis. The sample was passed through a reversed-phase liquid chromatography column (Welch Ultimate LP-C18, 4.6 mm × 250 mm, 5 μm) with a 30 °C column temperature, 1 mL/min flow rate, 10 μL injection, 95% 0.05 M ammonium formate (pH 4.5), a 5% methanol mixture mobile phase, and a 260-nm detection wavelength. The standard sample was used to determine the peak time and generate a standard curve for quantification.

### 2.7. Effect of Temperature, pH, Metal Ion Concentration and Reaction Time on ATP Synthesis

By using scaffold protein complex 1, we performed all the ATP synthesis reactions. A single variable was maintained to determine the effect of the optimum temperature, optimum pH, optimum substrate concentration, metal ions (Mg^2+^, K^+^, Na^+^, NH_4_^+^, Mn^2+^), enzyme ratio, and reaction time on the catalytic reaction in the ATP synthesis reaction. Each reaction gradient was set in three groups in parallel. After the reaction was completed, the reaction was terminated with a 1:30 volume ratio of 6 M hydrochloric acid, and the reaction product was diluted 6–10 times for detection.

### 2.8. Effect of Temperature, pH, Metal Ion Concentration and Substrate Concentration on SAM Synthesis

By using scaffold protein complex 2, we performed all the SAM synthesis reaction. A single variable was maintained to determine the effect of the optimum temperature, pH, metal ions (Mg^2+^ and K^+^), substrate concentration, and enzyme amount on the catalytic synthesis of SAM using MAT4 and MAT5. Each reaction gradient was set in three groups in parallel. After the reaction was completed, the reaction was terminated with a 1:30 volume ratio of 6 M hydrochloric acid, and the reaction product was diluted 6–10 times for detection.

When detecting the optimum temperature and pH, the other components were kept consistent in the reaction system. Reduced glutathione, MgCl_2_, KCl, l-Met, Tris-HCl (pH 8.0), and MAT4 or MAT5 was added to the system to provide ATP, followed by reactions at different temperatures and different pHs. After the reaction was terminated, the above method was repeated before detection.

## 3. Results 

### 3.1. Expression and Purification of Components of the Engineered Multidomain Scaffold Proteins

**Figure 3:**
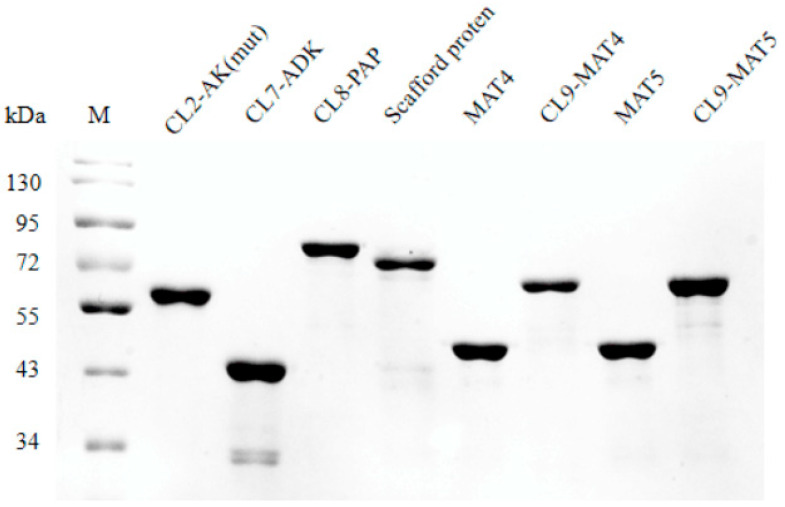
M: Protein marker, in which the theoretical molecular weight of the scaffold protein is approximately 67 kDa, CL2-AK (mut) is 55 kDa, CL7-ADK is 38.6 kDa, CL8-PAP is 75 kDa, MAT4 is 58 kDa, CL9-MAT4 is 59 kDa, MAT5 is 59 kDa, and CL9-MAT5 is 60 kDa.

In this study, we tried to construct multidomain scaffold proteins to synthesize ATP and SAM. Thus, the components of scaffold proteins should be obtained first. We synthesized the sequences of each component which was all subsequently subcloned into the plasmid pET23a(+), resulting in corresponding expression plasmids (pET28a-CL2-AK (mut), pET23a-CL7-ADK, pET23a-CL8-PAP, pET23a-MAT4, pET23a-MAT5, pET23a-CL9-MAT4, pET23a-CL9-MAT5, and pET23a-IM2-IM7-IM8-IM9). Then, the obtained recombinant plasmids were expressed in *E. coli* BL21 (DE3) or *E. coli* BL21PlysS, which were submitted to purification. SDS-PAGE of these target proteins is shown in Figure 3.

### 3.2. The Assembly of Scaffold Proteins

**Figure 4:**
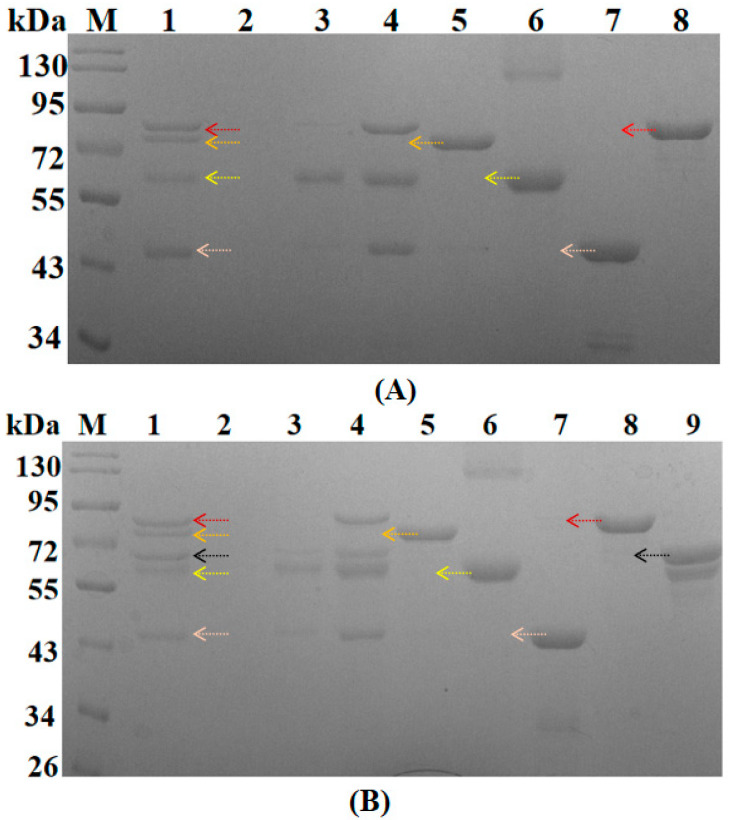
Multi-enzyme complex 1 (**A**) multi-enzyme complex 2 (**B**) and cellulose binding detection. (**A**) M is the protein marker; lane 1 is the cellulose bound to the multi-enzyme complex 1; lane 2 is the cellulose bound to the free enzyme; lane 3 is the flowthrough of lane 1; lane 4 is the flowthrough of lane 2; lane 5 is the scaffold protein; lane 6 is free CL2-AK (mut); lane 7 is free CL7-ADK; lane 8 is free CL8-PAP. The proteins of lane 1 are CL8-PAP, scaffold protein, CL2-AK (mut), and CL7-ADK from top to bottom; (**B**) M is a protein marker; lane 1 is cellulose bound to the multi-enzyme complex 2; lane 2 is cellulose bound to free enzyme; lane 3 is the flowthrough of lane 1; lane 4 is the flowthrough of lane 2; lane 5 is the scaffold protein; lane 6 is free CL2-AK (mut); lane 7 is free CL7-ADK; lane 8 is free CL8-PAP; lane 9 is free CL9 -MAT5. The proteins of lane 1 are CL8-PAP, scaffold protein, CL9-MAT5, CL2-AK (mut), and CL7-ADK from top to bottom.

When series CL-labeled proteins are bound to the CBM3 complex, SDS-PAGE can be used to detect the complex assembly status. The detection results are shown in Figure 4. It shows that the scaffold protein can fully assemble with each CL-labeled protein.

### 3.3. Optimum Reaction Temperature and pH for ATP Synthesis

**Figure 5:**
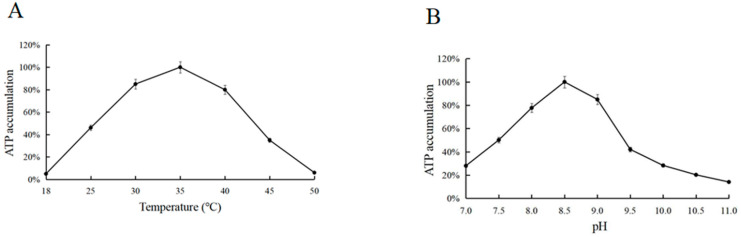
(**A**) Temperature optimization for ATP accumulation. (**B**) pH optimization for ATP accumulation.

Under a single variable, the gradient reaction at 18–50 °C showed that the optimum reaction temperature for ATP was 35 °C (Figure 5A). Similarly, the gradient reaction at pH 7–11 showed that the optimum reaction pH was 8.5. In the following experiments, the reactions were performed at 35 °C and pH 8.5 (Figure 5B).

### 3.4. Effect of the Concentration of Mg^2+^, K^+^, NH_4_^+^, Na^+^, Mn^2+^ and Tris-HCl on ATP Synthesis

**Figure 6:**
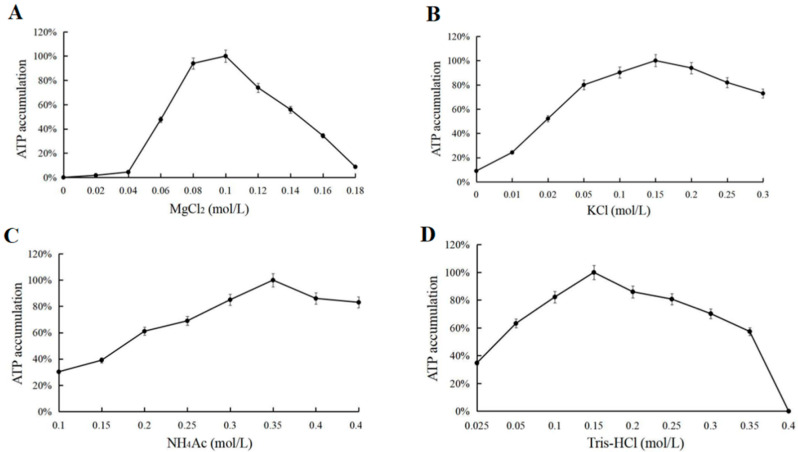
(**A**) MgCl_2_ optimization for ATP accumulation. (**B**) KCl optimization for ATP accumulation. (**C**) NH_4_Ac optimization for ATP accumulation. (**D**) Tris-HCl optimization for ATP accumulation.

A single variable was used to determine the effects of 0–0.18 M MgCl_2_, 0–0.3 M KCl, 0–0.45 M NH_4_Ac, 0–0.2 M MnCl_2_, 0–0.3 M NaCl, and 0.025–0.45 M Tris-HCl on ATP synthesis (Figure 6). Mg^2+^ was an essential ion for the ATP synthesis reaction and the optimum concentration to promote the reaction was 0.1 M. Other metal ions were not essential components for the reaction, but K^+^ and NH_4_^+^ promote the reaction and the optimum concentrations were 0.15 M and 0.35 M, respectively. Na^+^ and Mn^2+^ had an inhibitory effect on the reaction (results not shown). The optimum concentration of Tris-HCl was 0.15 M for pH buffering.

### 3.5. Effect of Adenosine and Sodium Hexametaphosphate on ATP Synthesis

**Figure 7:**
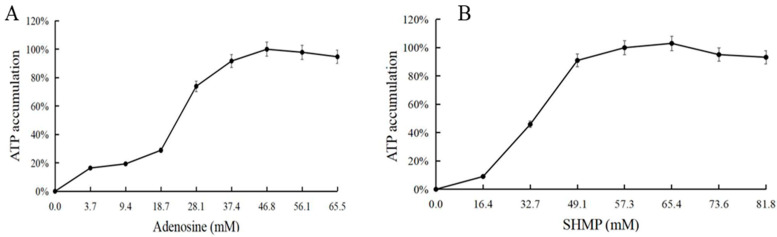
(**A**) Adenosine optimization for ATP accumulation. (**B**) SHMP optimization for ATP accumulation.

Adenosine and sodium hexametaphosphate (SHMP) were used as the substrates for ATP synthesis (Figure 7). Under a single variable, 0–17.5 g/L of adenosine and 0–50 g/L of sodium hexametaphosphate were used to set the gradient reaction. The consumption of adenosine and sodium hexametaphosphate were 12.5 g/L (46.8 mM) and 40 g/L (65.4 mM), respectively.

### 3.6. Effect of Reaction Time and Dosage Ratio of Multi-Enzyme Components on ATP Synthesis

**Figure 8:**
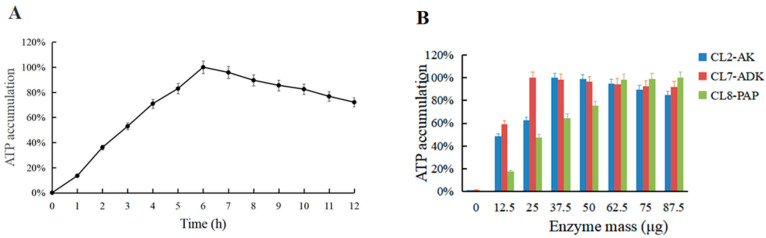
(**A**) Time optimization for ATP accumulation. (**B**) Enzyme mass optimization for ATP accumulation.

Under the above optimum conditions, a time gradient reaction was set with a 1 h interval. ATP synthesis was maximal at a 6 h reaction time (Figure 8A); however, the amount of ATP decreased when the time was extended because the ratio of the three enzyme components used in ATP synthesis was not optimum; thus, ATP produced other substances (ADP and AMP). Therefore, a single enzyme gradient reaction of 0–87.5 μg was set to determine the optimum enzyme ratio. CL2-AK (mut), CL7-ADK, and CL8-PAP had corresponding molar ratios of 1.1:1:1.3 in utilization of ATP accumulation (Figure 8B).

In summary, the optimum system for ATP synthesis included 0.15 M Tris-HCl (pH 8.5), 0.1 M MgCl_2_, 0.15 M KCl, and 0.35 M NH_4_Ac. At the corresponding enzyme dosage, molar ratios of 1.1:1:1.3, CL2-AK (mut), CL7-ADK, and CL8-PAP reacted with 40 g/L of sodium hexametaphosphate and 12.5 g/L of adenosine at 35 °C for approximately 6 h to produce approximately 25 g/L of ATP, 15 g/L of ADP, and 5 g/L of AMP.

### 3.7. Optimum Reaction Temperature and pH for MAT4 and MAT5

**Figure 9:**
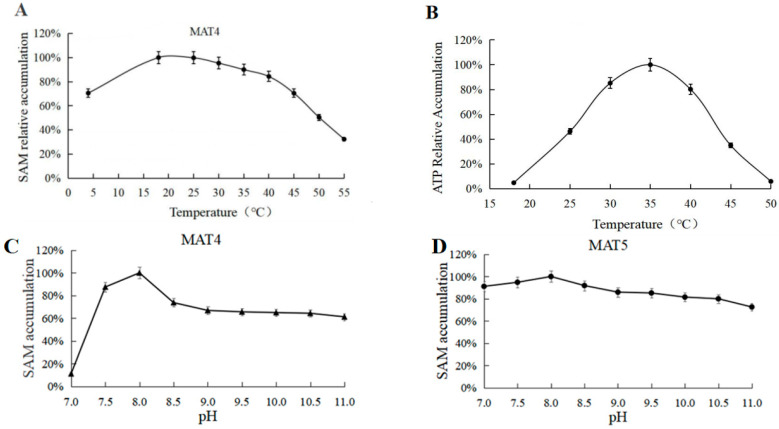
(**A**) Temperature optimization for SAM accumulation (MAT4). (**B**) Temperature optimization for SAM accumulation (MAT5). (**C**) pH optimization for SAM accumulation (MAT4). (**D**) pH optimization for SAM accumulation (MAT5).

Under a single variable, the gradient reaction at 4–55 °C showed that the optimum reaction temperature for MAT4 was 18 °C (Figure 9A), and the optimum reaction temperature for MAT5 was 35 °C (Figure 9B). The gradient reaction at pH 7–11 showed that the optimum pH for MAT4 and MAT5 was 8, and MAT4 activity was greatly inhibited when the pH was <7, while MAT5 had a wider pH range (Figure 9C,D).

### 3.8. Effect of Synchronous and Asynchronous Reactions on SAM Synthesis

SAM synthesis requires the participation of ATP. The ATP and SAM synthesis reactions were combined for the synthesis of SAM. Two means of combined action were investigated (synchronous and asynchronous reactions). The substrates and enzymes required by the two reaction systems in the same system for a one-step reaction is a synchronous reaction, whereas the product mixture and metal ions required for the synthesis of MAT and SAM as substrates to synthesize SAM after the ATP synthesis reaction is over is an asynchronous reaction. Due to the different ion concentration requirements of the two reactions, a synchronous reaction will inhibit ATP synthesis due to a high Mg^2+^ concentration, leading to inhibited SAM synthesis, or SAM synthesis will be inhibited due to a low Mg^2+^ concentration, resulting in less SAM accumulation (the effect of ion concentration on SAM synthesis in the synchronous reaction is not shown). Therefore, an asynchronous reaction favors SAM synthesis.

**Figure 10:**
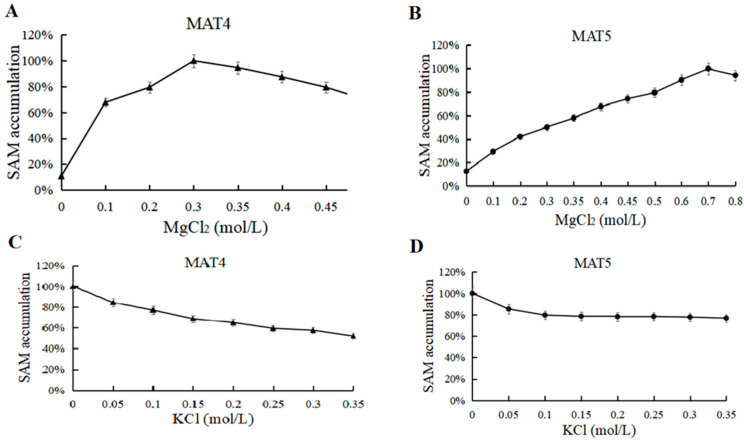
(**A**) MgCl_2_ optimization for SAM accumulation (MAT4). (**B**) MgCl_2_ optimization for SAM accumulation (MAT5). (**C**) KCl optimization for SAM accumulation (MAT4). (**D**) KCl optimization for SAM accumulation (MAT5).

As the MAT structure contains Mg^2+^ and K^+^ sites, the two ions are extremely critical to the reaction. Asynchronous reactions include two reactions (ATP and SAM synthesis). In the first reaction system, there is Mg^2+^ and K^+^ participation. Therefore, in the second step it is necessary to determine the appropriate reaction concentration. To test the reaction catalyzed by MAT4 and MAT5, 0–0.8 M MgCl_2_ and 0–0.4 M KCl were selected. MAT4 needed the addition of 0.7 M MgCl_2_ and MAT5 needed the addition of 0.3 M MgCl_2_ on the basis of the first reaction (Figure 10A,B); neither MAT4 nor MAT5 needed the addition of K^+^ (Figure 10C,D).

### 3.9. Effect of Catalytic Reaction Time on SAM Synthesis

**Figure 11:**
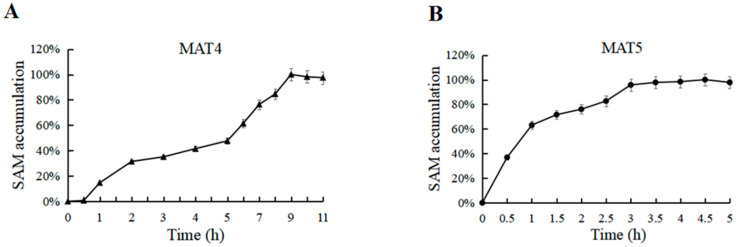
(**A**) Time optimization for SAM accumulation (MAT4). (**B**) Time optimization for SAM accumulation (MAT5).

MAT4 and MAT5 catalyze the production of SAM, but at different catalytic rates (Figure 11). With limited l-Met, when MAT4 was used to catalyze the production of SAM, the catalytic rate was slow and the maximum accumulation was reached in approximately 9 h. When MAT5 was used to catalyze the production of SAM, the rate was relatively fast, >50% of the total amount accumulated in approximately 30 min and the maximum accumulation was reached in approximately 3 h by converting an appropriate amount of substrate. In comparison, an equal amount of MAT5 had higher catalytic efficiency and produced more SAM by conversion.

### 3.10. Effect of l-Met and MAT on SAM Synthesis

**Figure 12:**
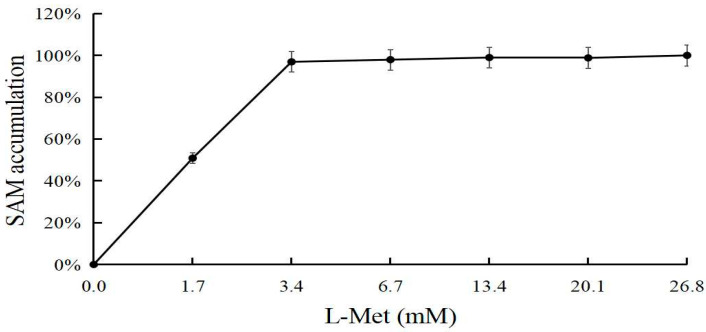
l-Met optimization for SAM accumulation.

**Figure 13:**
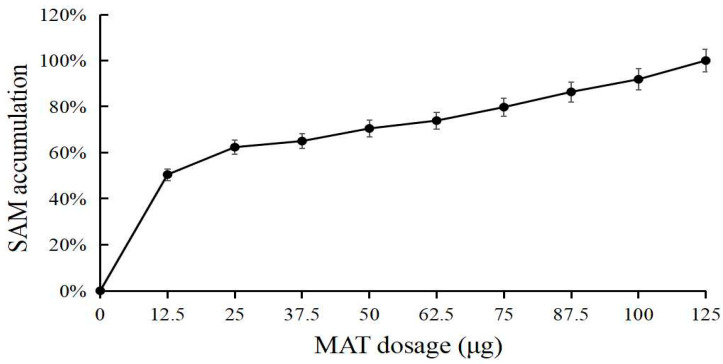
The effect of MAT dosage on SAM production.

When tested using a specific enzyme amount (25 μg), approximately 0.5 g/L of l-Met substrate was effectively converted, and the SAM yield was approximately 1.2 g/L. The synthesis rate of SAM was 25 g/L (Figure 12). When l-Met was sufficient, the amount of enzyme increased, and more SAM was synthesized. When the amount of enzyme was increased in the system, the yield of SAM increased slowly within a specific range, showing no obvious plateau (Figure 13).

## 4. Discussion

Enzymes can be combined sequentially in a single reactor to produce a cascade or domino reaction. In living cells, enzymes catalyze a variety of metabolic processes and involve multiple reaction steps. The efficient transfer of intermediate compound from one catalytic site to another is achieved by the formation of enzyme complexes. This kind of phenomenon has inspired researchers to artificially combine biocatalysts together. By adding multiple enzymes in the same reaction vessel, a multi-step enzymatic reaction could be performed in vitro [20,21,22,23]. Compared with the method of using the metabolic pathways in the microbial host, the reaction conditions are easy to control. In addition, the substrate can be freely selected, because there is no problem with the toxicity of the intermediate compound. Moreover, the final product is of very high purity in the system since there are no side reactions and cell metabolites.

In order to improve the multi-enzyme reaction rates and reaction efficiency, scaffold proteins are designed to combine multienzymes to produce functional metabolites through the substrate channels [23,26,27,28,29]. Scaffold proteins are not randomly aggregated but are usually used to organize multiple binding partners to promote their interaction and function [26,27,28,29]. Vassylyev et al. reported an efficient one-step ultra-high-affinity chromatography system for the purification of proteins [24,30]. The 16 kDa CL7 tag which was engineered from the *E. coli* Colicin E7 DNase (CE7) retains the ultra-high binding affinity with its inhibitor 10 kDa Immunity protein 7 (IM7). Inspired by this paper, we employed CL2/IM2, CL7/IM7, CL8/IM8 and CL9/IM9 to construct artificial scaffold proteins to achieve ATP and SAM synthesis. The IM2, IM7, IM8 and IM9 were fused with CBM3 by linkers. This complex could bind to cellulose. All the enzymes, including AK, ADK, PAP, MAT4 and MAT5, were fused with CL protein. When CBM3-IM2-IM7-IM8 or CBM3-IM2-IM7-IM8-IM9 protein and CL-labeled proteins were mixed, they could assemble as complex 1/complex 2 (Figure 1) which was subsequently used to produce ATP or SAM. Assembling experiments indicated that the CBM3 domain can fully assemble with each CL-labeled protein (Figure 3).

In previous studies, polyphosphate kinases were widely used to make the conversions among AMP, ADP and ATP. For example, adenosine kinase (ADK), two family-2 polyphosphate kinases (PPK2-I and PPK2-II) are combined to make an ATP regeneration system which was used to synthesize SAM [31,32]. In another research, an AMP regeneration system was constructed by the adenosine kinase from *Saccharomyces cerevisiae* (*Sc*ADK), a polyphosphate kinase from *Acinetobacter johnsonii* (*Aj*PPK2), and a polyphosphate kinase from *Sinorhizobium meliloti* (*Sm*PPK2). Then, this ATP regeneration system was used to produce 2′3′-cGAMP [33]. By using this ATP regeneration system, ATP need to be added to initiate the catalytic reaction. This makes the reaction complicated and increases the synthesis cost. In this study, we made a new ATP regeneration system which has not been reported before. This system does not need ATP to initiate the reaction. Two kinase (AK, ADK) and phosphate-adenylate phosphotransferase (PAP) were utilized. The optimal temperature and pH of these three enzymes are all 35 °C and 8.5, which were in agreement with the reaction conditions by using scaffold protein complex 1. Our complex 1 provides an attractive alternative to these conventional ATP regeneration systems. By applying complex 1, we achieved ATP production using cheap substrate adenosine and sodium hexametaphosphate.

After the ATP regeneration system was built, we added CL-labeled MAT into the system to synthesize SAM. The scaffold protein complex 2 successfully converted l-Met to SAM. Here, we provided two methionine adenosyltransferase MAT4 and MAT5 which could all achieve the SAM production. This SAM reaction conditions are simple and easy to control. The product is easy to extract. The catalytic reaction maintains a high substrate conversion rate.

All enzyme components of complex 1/2 are very easy to obtain by using an *E. coli* expression system. The recombinant proteins are easy to purify by using a Ni-NTA column. The reaction pH, temperature and other conditions of these enzymes are very friendly to industrial production. Here, ATP synthesis using inexpensive substrates (adenosine and sodium hexametaphosphate) significantly reduces the ATP production cost. The synthesized ATP is directly used for SAM synthesis, which also solves the problem of expensive ATP raw material for SAM synthesis and reduces environmental pollution. In addition, the enzymatic synthesis of ATP and SAM is simple and easy and provides an excellent solution for the production of SAM and a feasible alternative for other enzymatic reactions that require ATP.

## 5. Conclusions

In this study, we tried to produce ATP and SAM by engineered multidomain scaffold proteins. First, an artificial scaffold protein containing the CBM3 domain, IM proteins (IM2, IM7, IM8, IM9) and CL-labeled proteins (CL7-ADK; CL2-AK; and CL8-PAP) was assembled to form complex 1 for catalytic reactions to increase ATP production. This scaffold protein produced approximately 25 g/L of ATP with approximately 15 g/L of ADP and 5 g/L of AMP using 12.5 g/L of adenosine and 40 g/L of sodium hexametaphosphate reaction at 35 °C and a pH of 8.5 for 6 h. Based on the above ATP synthesis system, two CL-labeled methionine adenosyltransferases (CL9-MAT4 and CL9-MAT5) were applied to construct scaffold protein complex 2 to achieve SAM synthesis. Approximately 25 μg of MAT4 in a reaction system with 0.3 M MgCl_2_ at 20 °C and a pH of 8 catalyzed 0.5 g/L of l-Met to produce approximately 0.9 g/L of SAM. Approximately 25 μg of MAT5 with 0.7 M MgCl_2_ at 35 °C and a pH of 8 catalyzed 0.5 g/L of l-Met to produce approximately 1.2 g/L of SAM. Here, we showed that low-cost substrates can be efficiently converted into high-value additional ATP and SAM by engineered multidomain scaffold proteins.

## Data Availability

All relevant data of this study are presented. Additional data will be provided upon request.
